# Euploidy in somatic cells from R6/2 transgenic Huntington's disease mice

**DOI:** 10.1186/1471-2121-6-34

**Published:** 2005-09-13

**Authors:** Åsa Petersén, Ylva Stewénius, Maria Björkqvist, David Gisselsson

**Affiliations:** 1 Neuronal Survival Unit, Department of Experimental Medical Science, Wallenberg Neuroscience Center, Lund University, Sweden; 2 Department of Clinical Genetics, University Hospital, Lund, Sweden; 3 Unit of Molecular Metabolism, Division of Diabetes, Metabolism, and Endocrinology, Department of Experimental Medical Science, Lund University, Sweden

## Abstract

**Background:**

Huntington's disease (HD) is a hereditary neurodegenerative disorder caused by a CAG repeat expansion in the *HD *gene. The huntingtin protein expressed from *HD *has an unknown function but is suggested to interact with proteins involved in the cell division machinery. The R6/2 transgenic mouse is the most widely used model to study HD. In R6/2 fibroblast cultures, a reduced mitotic index and high frequencies of multiple centrosomes and aneuploid cells have recently been reported. Aneuploidy is normally a feature closely connected to neoplastic disease. To further explore this unexpected aspect of HD, we studied cultures derived from 6- and 12-week-old R6/2 fibroblasts, skeletal muscle cells, and liver cells.

**Results:**

Cytogenetic analyses revealed a high frequency of polyploid cells in cultures from both R6/2 and wild-type mice with the greatest proportions of polyploid cells in cultures derived from skeletal muscle cells of both genotypes. The presence of polyploid cells in skeletal muscle *in vivo *was confirmed by fluorescence *in situ *hybridisation with centromeric probes. Enlarged and supernumerary centrosomes were found in cultures from both R6/2 and wild-type mice. However, no aneuploid cells could be found in any of the tissues.

**Conclusion:**

We conclude that polyploid cells are found in fibroblast and skeletal muscle cultures derived from both R6/2 and wild-type littermate mice and that aneuploidy is unlikely to be a hallmark of HD.

## Background

Huntington's disease (HD) is a hereditary neurodegenerative disorder caused by a CAG repeat expansion in the *huntingtin *gene [1]. It is characterized by personality changes, motor disturbances, cognitive decline, and weight loss [2]. Neuropathologically, the disease is reflected by neurodegeneration, primarily in the neostriatum, the cerebral cortex and the hypothalamus, with the appearance of cytoplasmic and intranuclear aggregates of misfolded huntingtin in neurons [3]. In HD patients and transgenic models of the disease, wild-type (wt) and mutant huntingtin are expressed in most tissues [4]. Neither the pathogenetic mechanism of HD nor the normal function of huntingtin is fully understood. Huntingtin interacts with key players of the mitotic machinery, such as the microtubuli and the centrosomes [5]. Interestingly, when huntingtin was identified in 1993 [1], one of its few known sequence motifs with established functions was the HEAT repeats [6]. These sequences are also found in proteins involved in mitotic progression and chromosomal dynamics [7]. The R6/2 transgenic mouse is the most widely used model to study HD. It expresses exon 1 of the human HD gene and displays several features of HD such as motor dysfunction, neuronal huntingtin aggregates, weight loss, and premature death at around 14 weeks of age [8]. In fibroblast cultures derived from R6/2 mice, a reduced mitotic index and high frequencies of cells with multiple centrosomes and aneuploidy have recently been reported [9]. Aneuploidy is characteristic for neoplastic cells, cells exposed to carcinogens, and cells from cancer-prone patients with hereditary chromosome instability syndromes. However, the phenomenon has rarely, if ever, been described in neurodegenerative disease in the absence of an increased risk for neoplasia. To further explore this unexpected aspect of HD, we have now performed extensive cytogenetic analyses of fibroblasts, skeletal muscle cells, and liver cells from 6- and 12-week-old R6/2 and wt littermate mice. We found no evidence of aneuploidy in R6/2 cells, but a suprisingly high frequency of polyploid cells in cultures derived from both R6/2 and wt littermate mice.

## Results

### Cytogenetic analysis

**Figure 1:**
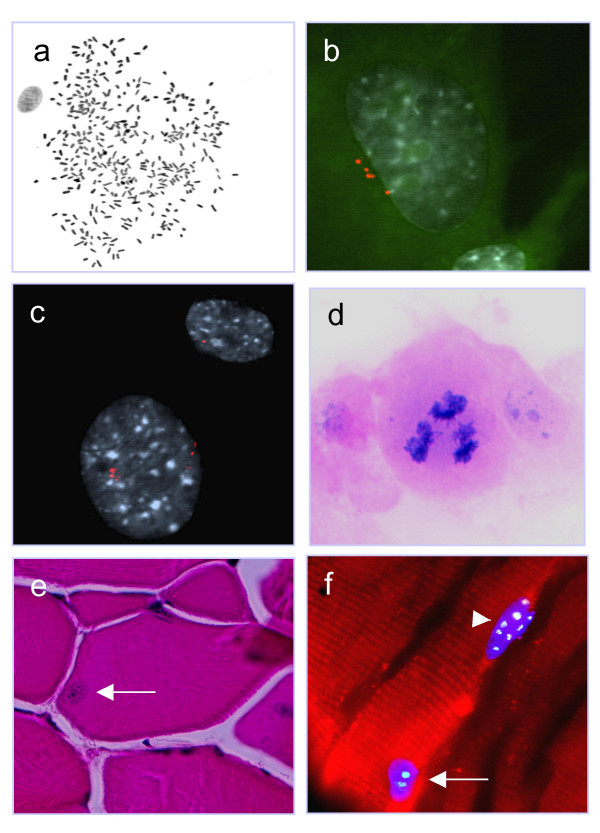
**Cytogenetic and immunofluorescent analysis. **G-banded polyploid metaphase cell from wt31 abdominal muscle (a). Multiple centrosomes (red) in cells from the peritoneal fat of R6/2 78 (b; green autofluorescence indicates cytoplasm) and from the abdominal muscle of wt77 (c; autofluorescence removed for clarity). Tripolar anaphase cell visualized by haematoxylin-eosin staining in a cell (passage 1) from the abdominal muscle of wt30 (d). Large nucleus (arrow) in a skeletal muscle fibre from wt77 (e). One nucleus with two chromocenters (arrow; green) and one with eight chromocenters (arrowhead) in a skeletal muscle fibre from wt76 (f).

Fresh biopsies were taken from ear lobes, abdominal skeletal muscles, peritoneal fat, and liver from sacrificed six- and 12-week-old mice (Table 1). Cells from R6/2 and wt littemate mice grew equally well in culture up to five passages, after which the culture process was terminated. Analysis of metaphase peritoneal fat fibroblasts, skeletal muscle cells, and liver cells from 6-week-old R6/2 and wt littermate mice showed a normal diploid chromosome complement in the vast majority of cells and a tetraploid chromosome number in 3–32% of the cells. Cells taken from the abdominal skeletal muscle and the liver also showed 2–19% polyploid cells, with chromosome numbers up to the decaploid level (N = 400; Fig. 1a). These polyploid cells were present in biopsies both from R6/2 and wt mice. The highest frequencies were found in abdominal muscle biopsies from wt animals. After *in vitro *propagation up to four passages of muscle cells from one wt and two R6/2 mice, the frequency of polyploid cells had increased, compared to the first passage, from 8% to 45% in the wt culture, including cells with up to 600 chromosomes, whereas the frequency of such cells had decreased in one of the R6/2 cultures and was largely unchanged in the other. No more than four aneuploid cells were found in any of the cultures. These invariably showed loss of only one or two chromosomes compared to the diploid or tetraploid levels, or loss of up to four chromosomes compared to polyploid levels. In skeletal muscle cells, ear lobe fibroblasts, and peritoneal fat fibroblasts from 12-week-old animals, similar results were obtained. No more than four aneuploid cells (including hypodiploid, hypotetraploid, and hypopolyploid cells) were detected in any of the week-12 cultures.

**Table 1:** Cytogenetic data.

Mouse number	Biopsy site	Passage number	Ploidy < 2n	Ploidy = 2n	Ploidy 2n-4n	Ploidy = 4n	Ploidy > 4n	Total number of cells analyzed	Polyploid cells (%)^a^
***Wild-type week 6***									
30	AM	1	1	29	1	11	10	52	19
31	PF	1	2	46	0	11	0	59	0
31	AM	1	1	26	0	19	4	50	8.0
31	AM	4	0	3	0	3	5	11	45
									
***Wild-type week 12***									
76	EL	1	2	46	0	4	0	52	0
76	EL	5	0	50	0	17	0	67	0
76	PF	1	0	42	0	7	1	50	2.0
76	AM	1	0	10	0	1	1	12	8.3
77	EL	1	0	60	0	4	0	64	0
77	EL	5	0	22	3	20	5	50	10
77	EL	1	0	46	0	5	1	52	19
77	AM	1	0	85	0	30	2	117	1.7
									
***R6/2 week 6***									
									
38	PF	1	0	45	0	5	0	50	0
38	AM	1	0	39	0	12	5	56	8.9
38	AM	4	0	32	0	32	1	65	1.5
38	LC	1	0	24	2	17	7	50	14
39	AM	1	0	33	1	12	4	50	8.0
39	AM	4	0	25	0	30	3	58	5.2
									
***R6/2 week 12***									
78	EL	1	1	50	0	4	0	55	0
78	EL	5	0	39	1	19	3	62	4.8
78	PF	1	0	53	0	3	0	56	0
78	AM	1	0	30	0	4	1	35	2.9
79	PF	1	0	70	1	7	0	78	0
79	AM	1	0	50	1	7	0	58	0

^a ^Cells with a ploidy level above the tetraploid (4n). AM, abdominal muscle cells; PF, peritoneal fat fibroblasts; EL, ear lobe fibroblasts; LC, liver cells.

### Centrosome morphology and mitotic polarity

Centrosome detection was performed on cultured skeletal muscle cells, peritoneal fat fibroblasts, and ear lobe fibroblasts (passage 1 and also passage 5 of the ear lobe biopsies) from 12-week-old mice. This revealed abnormally enlarged centrosomes (> 2 centrioles) in a small population of cells in all cases (1–4%). A proportion of these cells (0.5–2% out of all cells) had three or four centrosomes, whereas the remaining cells had normal centrosome numbers (one or two; Fig 1b and 1c). There were no significant differences between wt and R6/2 cells (p > 0.05; > 100 cells analysed from each culture). Because supernumerary centrosomes have been associated with spindle multipolarity at mitosis, we also analysed cell division figures from the cultured biopsies of 6-week and 12-week mice after haematoxylin-eosin staining. Tripolar metaphase and anaphase configurations were detected in cells from the muscle biopsies from one of the 6-week old R6/2 mice and one of the 6-week old wt mice (2/160 cells and 2/232 cells respectively; Fig. 1d) but not in cells from any of the other biopsies (>150 cells analysed per biopsy).

### Identification of polyploid cells in vivo

In both R6/2 and wt mice, the highest rate of polyploidy was noted in cultures established from the abdominal muscle. To corroborate these findings *in vivo *and to identify the cell type harbouring an abnormally high chromosome number, 10 μm tissue sections from two wt and two R6/2 mice were first examined after haematoxylin-eosin staining. This revealed a low frequency (1–2%) of nuclei with diameters three to four times the normal range residing in the striated muscle fibres (Fig. 1e). Such enlarged nuclei were neither found in the adjacent fibrous tissue, nor in the vascular tissue penetrating the muscles. To exclude that these nuclei were simply artifacts caused by oblique cutting of the sections, fluorescence *in situ *hybridization (FISH) with a murine pan-centromeric probe was performed on muscle tissue sections from one wt mouse. As expected, this revealed two chromocenters in the majority of nuclei (536/788 = 68%) and small populations of nuclei with one (10%), three (9%), or four (9%) chromocenters. These signal configurations were also found in the adjacent fibrous tissues. However, there was also a small population (4%) of large nuclei with seven or eight chromocenters (Fig. 1f), all of which were present in striated muscle fibres. Nuclei with > 4 chromocenters were also found in abdominal muscle fibres from the other 12-week-old wt mouse and the two R6/2 mice, in proportions of 1–4%.

## Discussion

The function of normal huntingtin is not fully elucidated. Sequences of known functions within huntingtin include HEAT repeats, which are involved in chromosome dynamics [7]. In HD, the expanded polyglutamine in huntingtin is thought to alter its protein interactions [5]. A previous study described high frequencies of multiple centrosomes and aneuploidy in fibroblast lines derived from R6/2 mice and HD patients [9]. Aneuploidy is closely connected to neoplastic disease, as the vast majority of tumours exhibit acquired chromosome abnormalities [10]. Moreover, most inherited chromosome instability syndromes are associated with a significantly elevated risk of cancer. However, the risk of malignant tumours has been shown to be lower in HD patients than in the normal population [11]. To explore this ostensibly paradoxical situation, we performed cytogenetic analyses of cultured cells from multiple tissues in R6/2 mice. Although a high number of cells were analysed, we could find only minute populations of anueploid cells, occurring at similar frequencies in wt and R6/2 mice. These were typically hypodiploid, hypotetraploid, or hypopolyploid with loss of only few chromosomes, indicating that they were most probably due to artifactual loss of chromosomes during preparation. Hence, we found no convincing evidence for aneuploidy. The reason for the discrepancy of results between our study and the previous report [9] may arise from differences in the genetic background of the R6/2 colonies, different culture procedures, or differences in the methods for chromosome preparation. Furthermore, in our study, we defined aneuploidy according to the ISCN (1995) recommendations [12], whereas the previous study defined aneuploid cells as any cells harboring a chromosome number different than 2n = 40. Some of the cells scored as polyploid in our study might thus have been scored as "aneuploid" in the previous study by Sathasivam et al [9]. We found small populations of highly polyploid cells in liver, fibroblast and skeletal muscle cultures derived from R6/2 and wt littermate mice. In these cultures, derived from two mice per genotype, there was no difference in the frequency of polyploid cells between R6/2 and wt mice.

Polyploid cells have been found in several murine tissue including vascular smooth muscle [13], ovaries [14], cardiomyocytes [15], colonic fibroblasts [16], liver cells, bladder epithelial cells, uterine decidua cells, trophoblasts, and megakaryocytes [17]. Our study supports that polyploidy is a normally occurring phenomenon in many murine tissues and shows that skeletal muscle cells may have an unusually high frequency of polyploid cells. An earlier investigation describes a high frequency of multiple centrosomes in fibroblast cell lines from R6/2 mice and from HD patients [9]. In the present study, we found low frequencies of cells with multiple centrosomes in cultures from both R6/2 and wt littermate mice. In neoplastic cells, supernumerary centrosomes are associated with multi-polar cell divisions and have traditionally been suggested to generate aneuploid daughter cells [18]. Somewhat surprisingly, we found multipolar mitoses at a low frequency in skeletal muscle cultures from both genotypes. To our knowledge, this type of mitotic aberration has not been shown previously in non-neoplastic cells. Whether similar mitotic aberrations also occur in murine skeletal muscle *in vivo *remains to be established.

## Conclusion

The present study shows that even though polyploidy may occur to a similar extent in wt and R6/2 cultured somatic cells, aneuploidy does not occur at a high frequency in R6/2 cells.

## Methods

### Transgenic mice

Transgenic HD mice of the R6/2 line were originally purchased from Jackson Laboratories (Bar Harbor, ME, USA) and the colony was maintained by breeding heterozygous R6/2 males with females from their background strain (F1 of CBA × C57Bl/6). Tails of the offspring were used to obtain DNA for determination of the genotype using a polymerase chain reaction assay [8]. The mice exhibit around 150 CAG repeats in the exon 1 of the *HD *gene. The mice were housed in groups with *ad libitum *access to food and water at a 12 h light/dark cycle and sacrificed at either 6 or 12 weeks of age using halothane anaesthesia. The experimental procedures were approved by the Ethical Committee at Lund University.

### Cell culture and morphological analyses

Cells were cultured in RPMI 1640 medium, supplemented with 17% bovine serum, glutamine, and antibiotics. Chromosome preparation, G-banding, analysis of mitotic morphology, haematoxylin-eosin staining, and FISH were performed according to standard methods [19]. The number of metaphase cells analyzed from each biopsy, genotype, and age are specified in Table 1. Mouse major satellite sequences were detected in at least 500 interphase nuclei per biopsy using commercially available probes (Cambio, Cambridge, UK). Centrosome morphology was visualized by immuno-fluorescence using a monoclonal anti-γ-tubulin antibody (1:1000, GTU-88, Sigma, St. Louis, MS). At least 100 cells from each biopsy were analysed.

## List of abbreviations

FISH fluorescence *in situ *hybridization

HD Huntington's disease

Wt wild-type

## Authors' contributions

ÅP participated in the design of the study as well as the writing of the manuscript. YS carried out the cell culturing and the chromosome preparation. MB prepared the muscle tissue. DG performed the cytogenetic and FISH analyses and participated in the design of the study, and the writing of the manuscript.

## References

[B1] Huntington's Disease Collaborative Research Group (1993). A novel gene containing a trinucleotide repeat that is expanded and unstable on Huntington's disease chromosomes. Cell.

[B2] Petersen Å, Brundin P (2002). Huntington's disease: the mystery unfolds?. Int Rev Neurobiol.

[B3] DiFiglia M, Sapp E, Chase KO, Davies SW, Bates GP, Vonsattel JP, Aronin N (1997). Aggregation of huntingtin in neuronal intranuclear inclusions and dystrophic neurites in brain. Science.

[B4] Landles C, Bates GP (2004). Huntingtin and the molecular pathogenesis of Huntington's disease. Fourth in molecular medicine review series. EMBO Rep.

[B5] Li SH, Li XJ (2004). Huntingtin-protein interactions and the pathogenesis of Huntington's disease. Trends Genet.

[B6] Andrade MA, Bork P (1995). HEAT repeats in the Huntington's disease protein. Nat Genet.

[B7] Neuwald AF, Hirano T (2000). HEAT repeats associated with condensins, cohesins, and other complexes involved in chromosome-related functions. Genome Res.

[B8] Mangiarini L, Sathasivam K, Seller M, Cozens B, Harper A, Hetherington C, Lawton M, Trottier Y, Lehrach H, Davies SW, Bates GP (1996). Exon 1 of the HD gene with an expanded CAG repeat is sufficient to cause a progressive neurological phenotype in transgenic mice. Cell.

[B9] Sathasivam K, Woodman B, Mahal A, Bertaux F, Wanker EE, Shima DT, Bates GP (2001). Centrosome disorganization in fibroblast cultures derived from R6/2 Huntington's disease (HD) transgenic mice and HD patients. Hum Mol Genet.

[B10] Mitelman F, Johansson B, Mertens F (2005). Mitelman Database of Chromosome Aberrations in Cancer. http://cgap.nci.nih.gov/Chromosomes/Mitelman.

[B11] Sorensen SA, Fenger K, Olsen JH (1999). Significantly lower incidence of cancer among patients with Huntington disease. Cancer.

[B12] Mitelman F, ed (1995). An International System for Human Cytogenetic Nomenclature.

[B13] Jones MR, Ravid K (2004). Vascular smooth muscle polyploidization as a biomarker for aging and its impact on differential gene expression. J Biol Chem.

[B14] Keighren M, West JD (1993). Analysis of cell ploidy in histological sections of mouse tissues by DNA-DNA in situ hybridization with digoxigenin-labelled probes. Histochem J.

[B15] Brodsky VY, Delone GV, Tsirekidze NN (1985). Genome multiplication in cardiomyocytes of fast- and slow-growing mice. Cell Differ.

[B16] Neal JV, Potten CS (1981). Polyploidy in the murine colonic pericryptal fibroblast sheath. Cell Tissue Kinet.

[B17] Keighren MA, Macfadyen LP, Hill AS, Patek CE, Telfer EE, West JD (2003). Polyploid cells in the mouse ovary. J Anat.

[B18] Hansemannn D (1891). Ueber patologische Mitosen. Arch Pathol Anat Phys Klin Med.

[B19] Gisselsson D, Jonson T, Yu C, Martins C, Mandahl N, Wiegant J, Jin Y, Mertens F, Jin C (2002). Centrosomal abnormalities, multipolar mitoses, and chromosomal instability in head and neck tumours with dysfunctional telomeres. Br J Cancer.

